# The SMAC mimetic, LCL-161, reduces survival in aggressive MYC-driven lymphoma while promoting susceptibility to endotoxic shock

**DOI:** 10.1038/oncsis.2016.26

**Published:** 2016-04-04

**Authors:** A C West, B P Martin, D A Andrews, S J Hogg, A Banerjee, G Grigoriadis, R W Johnstone, J Shortt

**Affiliations:** 1 Gene Regulation Laboratory, Peter MacCallum Cancer Centre, East Melbourne, VIC, Australia; 2 Hudson Institute of Medical Research, Clayton, VIC, Australia; 3 Department of Molecular and Translational Sciences, Monash University, Clayton, VIC, Australia; 4 Department of Microbiology & Immunology, Central Clinical School, Monash University, Clayton, VIC, Australia; 5 Sir Peter MacCallum Department of Oncology, University of Melbourne, Parkville, VIC, Australia; 6 Centre for Inflammatory Diseases, Monash University, Clayton, VIC, Australia; 7 Alfred Pathology Service, Alfred Health, Prahran, VIC, Australia; 8 Monash Haematology, Monash Health, Clayton, VIC, Australia; 9 School of Clinical Sciences, Monash Health, Monash University, Clayton, VIC, Australia

## Abstract

Inhibitor of apoptosis proteins (IAPs) antagonize caspase activation and regulate death receptor signaling cascades. LCL-161 is a small molecule second mitochondrial activator of caspase (SMAC) mimetic, which both disengages IAPs from caspases and induces proteasomal degradation of cIAP-1 and -2, resulting in altered signaling through the NFκB pathway, enhanced TNF production and sensitization to apoptosis mediated by the extrinsic pathway. SMAC mimetics are undergoing clinical evaluation in a range of hematological malignancies. Burkitt-like lymphomas are hallmarked by a low apoptotic threshold, conveying sensitivity to a range of apoptosis-inducing stimuli. While evaluating LCL-161 in the Eμ-*Myc* model of aggressive Burkitt-like lymphoma, we noted unexpected resistance to apoptosis induction despite ‘on-target' IAP degradation and NFκB activation. Moreover, LCL-161 treatment of lymphoma-bearing mice resulted in apparent disease acceleration concurrent to augmented inflammatory cytokine-release in the same animals. Indiscriminate exposure of lymphoma patients to SMAC mimetics may therefore be detrimental due to both unanticipated prolymphoma effects and increased susceptibility to endotoxic shock.

## Introduction

Inhibitor of apoptosis proteins (IAPs) (X-linked IAP, cIAP1 and cIAP2) possess baculoviral IAP repeat (BIR) domains that mediate binding to post-mitochondrial caspases.^1^ Mitochondrial permeabilization releases second mitochondrial activator of caspases (SMAC), which competes for BIR occupancy on IAPs to augment apoptosis induction. Accordingly, the initial basis for development of small molecule SMAC mimetics as antineoplastics was as simple proapoptotic agents. It was subsequently demonstrated that IAP antagonists induce proteasomal degradation of cIAP1 and cIAP2, enhancing both canonical and noncanonical NFκB signaling downstream of tumor necrosis factor (TNF) family receptors concurrent to the initiation of autocrine death receptor (DR) signaling.^2, 3^ Susceptible cell lines are exquisitely sensitive to IAP antagonists due to feedback amplification of the extrinsic apoptotic pathway, mediated primarily by TNFα.

LCL-161 (Novartis, Basel, Switzerland) is an orally available IAP antagonist with preclinical activity as a single agent demonstrated in multiple myeloma,^4^ glioblastoma^5^ and sarcoma.^5, 6^ In the absence of single-agent activity, LCL-161 sensitizes to apoptosis induction by chemotherapy or BCL-2 inhibition in hepatocellular carcinoma^7, 8^ and radiotherapy in esophageal carcinoma.^9^ Synergistic activation of the extrinsic apoptotic pathway was also demonstrated by combining LCL-161 with adenovirally-vectored TNFα in melanoma.^10^ The results of a phase I dose escalation study were recently reported.^11^ Despite biomarkers on *in vivo* cIAP1 degradation and cytokine release at well-tolerated doses, no objective responses were observed in the solid organ tumor setting. However, clinical trials with LCL-161 and other SMAC mimetics are ongoing, including in hematological malignancies such as multiple myeloma and acute myeloid leukemia.

Lymphomas driven by the *cMYC* oncogene are remarkable for high rates of basal proliferation and apoptosis. cIAP1 potentiates MYC activity, by ubiquitinating its negative regulator, MXD1.^12^ We therefore hypothesized that LCL-161 would show potent activity in the Eμ-*Myc* model of aggressive lymphoma, which is sensitive to a range of novel apoptosis-inducing stimuli.^13, 14, 15, 16^ Unexpectedly, Eμ-*Myc* lymphomas were highly resistant to LCL-161-induced apoptosis *in vitro*, despite engagement of IAP degradation and NFκB activation at ‘on-target' concentrations. Moreover, LCL-161 did not sensitize Eμ-*Myc* lymphomas to death-receptor-induced apoptosis. Interestingly, LCL-161 treatment of lymphoma-bearing mice accelerated disease progression culminating in a survival disadvantage compared with vehicle-treated controls. Analogous to the cytokine release syndrome (CRS) encountered in human trials,^11^ LCL-161 markedly exacerbated inflammatory cytokine-release following lipopolysaccharide (LPS) challenge. Thus, LCL-161 accelerates Eμ-*Myc* lymphoma and predisposes to septic shock *in vivo*. These findings mandate caution during the clinical evaluation of SMAC mimetics when used as single agents in hematological malignancies.

## Results and discussion

### LCL-161 induces cIAP1 degradation and NFκB activity in Eμ*-Myc* lymphoma

**Figure 1:**
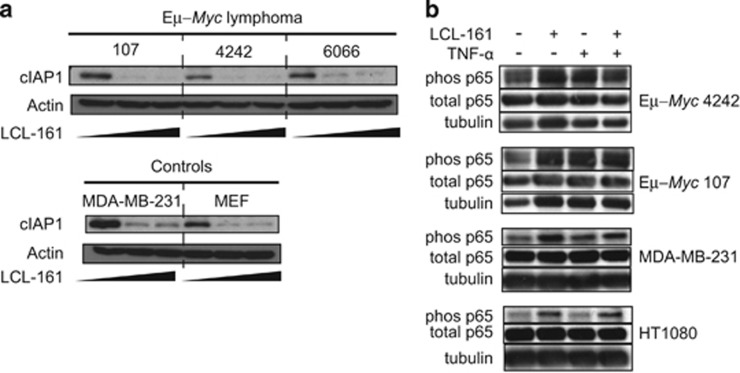
LCL-161 induces cIAP degradation and NFκB at on-target concentrations *in vitro*. (**a**) C57Bl/6-derived Eμ-*Myc* lymphomas (107, 4242 and 6066 derived and maintained as described previously^13^) as well as MDA-MB-231 and MEF control cells (cultured in DMEM with 10% heat-inactivated fetal calf serum (FCS), 2 mm L-glutamine and penicillin (100 U/ml)/streptomycin (100  μg/ml; all Gibco, ThermoFisher Scientific, Waltham, MA, USA at 37 °C and 5% CO_2_) were treated with increasing doses of LCL-161 (0.2 or 2 μM for 24 h; kindly provided by Novartis) or vehicle control. cIAP1 expression was assessed on protein lysates by western blot using standard techniques and antibodies against cIAP1 (#4952; Cell Signaling Technology, Danvers, MA, USA) and actin (#A2228; Sigma-Aldrich, St Louis, MO, USA) to confirm equivalent loading. (**b**) Eμ-*Myc* (4242 and 107) lymphomas, MDA-MB-231 and HT1080 cells were treated with LCL-161 (2 μM) and/or TNFα (2.5 ng/ml; R&D Biosystems, Minneapolis, MN, USA) for 2 h, and NFκB activation assessed by western blotting using antibodies against phosphorylated (Ser536) p65 (93H1 #3033; Cell Signaling Technology), total p65 (#ab7970; Abcam, Cambridge, UK) and then reprobed for tubulin (#T5326; Sigma-Aldrich) to confirm equivalent loading. Images shown are representative of three biological replicates.

IAP antagonists reportedly induce proteasomal degradation of cIAP1 and cIAP2, enhancing both canonical and noncanonical NFκB signaling downstream of TNF family receptors concurrent to the initiation of autocrine DR signaling.^2, 3^ We first investigated the capacity of LCL-161 to induce cIAP1 degradation *in vitro*. cIAP1 was readily degraded in three independently-derived Eμ-*Myc* lymphomas following the 24 h LCL-161 treatment at low concentrations (0.2–2 μM). Similar levels of cIAP1 degradation were observed in human breast cancer cells (MDA-MB-231) and mouse embryo fibroblasts (MEF; Figure 1a). Next, we investigated whether cIAP1 degradation induced downstream NFκB activation in Eμ-*Myc* cells. Upon treatment with LCL-161 or stimulation with TNFα, the NFκB subunit p65 was phosphorylated in Eμ-*Myc* lymphomas (Figure 1b). Similar phosphorylation of p65 in response to LCL-161 was observed in additional nonhematopoietic cancer cell lines (MDA-MB-231 and HT1080), even though these were relatively insensitive to TNFα stimulation (Figure 1b). Thus, Eμ-*Myc* lymphoma exhibited degradation of cIAP1 and NFκB pathway activation in response to LCL-161 *in vitro.*

### Eμ*-Myc* lymphoma is resistant to apoptosis induction by LCL-161

**Figure 2:**
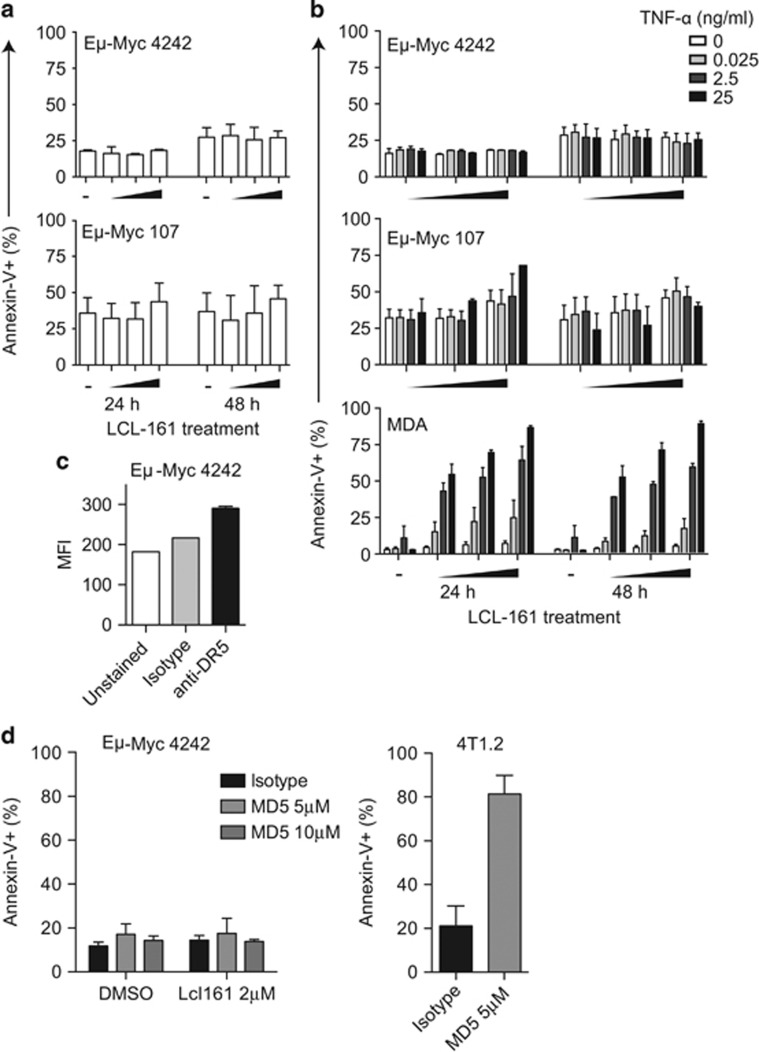
Eμ-*Myc* lymphomas are refractory to apoptosis via LCL-161 and the extrinsic apoptosis pathway. (**a**) Eμ-*Myc* lymphomas (4242 and 107; 1−10 × 10^5^) were treated with LCL-161 (0.2, 2 or 20 μM) or vehicle control (−) and assessed for apoptosis by flow cytometry at 24 and 48 h using Annexin-V (#17-8007; eBiosciences, San Diego, CA, USA) and viability dye (Fluoro-Gold; Fluorochrome, LLC, Denver, CO, USA) staining. (**b**) Eμ-*Myc* lymphomas (4242 and 107) and MDA-MB-231 cells (MDA) were treated with LCL-161 or vehicle control (as per **a**) in the presence of increasing doses of TNFα and assessed for apoptosis by flow cytometry after 24 or 48 h using Annexin-V and viability dye staining as described in **a**. (**c**) The surface expression of DR-5 on live Eμ-*Myc* 4242 cells was assessed by flow cytometry using an anti-DR-5 antibody (MD5-1; #119905; BioLegend, San Diego, CA, USA) or isotype control (HTK888; 400907; BioLegend) and viability dye staining. (**d**) Eμ-*Myc* 4242 (left panel) or 4T1.2 (right panel) cells were treated with plate-bound (8-well Protein A-coated strips; ThermoFisher, Waltham, MA, USA) MD5.1 at the concentrations indicated or isotype control (10 μM; UC8-1B9; both prepared as previously described)^31^ alone or in combination with LCL-161 (2 μM). Apoptosis was assessed after 24 h treatment by flow cytometry using Annexin-V and viability dye staining as described in **a**. Error bars represent the mean±s.e.m. of at least three independent experiments.

Similar to human MYC-driven lymphomas, Eμ-*Myc* lymphoma is sensitive to apoptosis induction by a range of novel and conventional therapeutics.^13, 14, 15, 16^ We therefore expected IAP degradation to correlate with a potent apoptotic response following the LCL-161 treatment. However, despite biochemical evidence of LCL-161-mediated cIAP1 degradation (Figure 1a), Eμ-*Myc* lymphoma cells were resistant to apoptosis induction by LCL-161 at corresponding concentrations and even with a 100-fold dose escalation (Figure 2a).

Apoptosis induction by IAP antagonists is primarily mediated by autocrine DR signaling via TNFα.^2, 3^ We therefore assessed the capacity of Eμ-*Myc* lymphoma cells to either secrete or respond to TNFα in response to LCL-161. TNFα was not detected upon assessment of the supernatant of LCL-161-treated Eμ-*Myc* cells by cytokine bead array (data not shown). However, exogenous TNFα induced p65 phosphorylation indicating intact TNF receptor activity (Figure 1b). We therefore assessed the capacity for LCL-161 to augment an apoptotic response to exogenous TNFα. Eμ-*Myc* cells were treated with LCL-161 in the presence of increasing TNFα concentrations (Figure 2b). Neither TNFα alone, nor the addition of TNFα to LCL-161 resulted in apoptosis of Eμ-*Myc* lymphoma cells, despite robust apoptosis induction in the MDA-MB-231 (LCL-161-sensitive control) line treated concurrently (Figure 2b).

### Eμ-*Myc* lymphoma is resistant to apoptosis induction by DR agonists

Given the lack of TNFα-induced cell death in Eμ-*Myc* cells, we next sought to determine if Eμ-*Myc* cells were capable of undergoing apoptosis in response to direct stimulation of the extrinsic apoptosis pathway. Surface expression of DR-5 was detected on Eμ-*Myc* lymphoma cells, albeit in low levels (Figure 2c). However, Eμ-*Myc* lymphoma cells were resistant to the DR-5 agonistic antibody, MD5.1, administered alone or in combination with LCL-161 (Figure 2d). In contrast, MD5.1 readily induced apoptosis in the 4T1.2 murine breast cancer cell line. Together these data suggest Eμ-*Myc* lymphoma is resistant to LCL-161, despite cIAP1 degradation and NFκB engagement due to insensitivity to extrinsic apoptotic stimuli, and a subsequent failure to engage autocrine DR signaling described in other models.^2, 3^

### LCL-161 reduces the survival of mice bearing Eμ-*Myc* lymphoma

The absence of an *in vitro* apoptotic response to LCL-161 does not necessarily preclude *in vivo* activity. For example, non-tumor cell autonomous mechanisms of action including paracrine signaling, microenvironmental changes and the host immune response may be important mediators of therapeutic responses in the absence of direct apoptosis induction. Indeed, we have previously demonstrated that an intact host immune system is required for optimal responses to other novel antilymphoma agents *in vivo.*^17^

**Figure 3:**
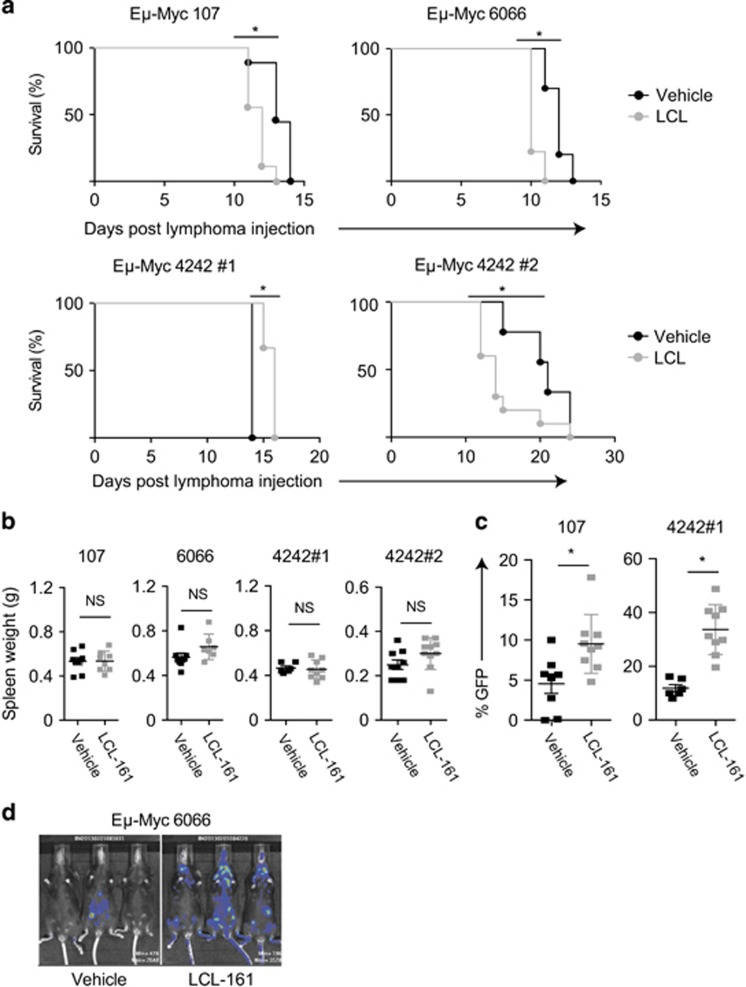
LCL-161 accelerates the progression of Eμ-*Myc* lymphoma *in vivo*. (**a**) Eμ-*Myc* lymphoma cells were endowed with either GFP or GFP and luciferase expression by routine retroviral techniques using the MSCV-IRES-GFP and MSCV-IRES-Luc vectors, respectively.^32^ Next, 5−10 × 10^5^ Eμ-*Myc* lymphoma cells 107/MSCV.GFP (*n*=9 per group; left upper panel), 6066/MSCV.GFP-Luc (*n=*10 vehicle, *n*=9 LCL-161; right upper panel), 4242/MSCV.GFP, expt #1 (*n*=6 vehicle*; n*=9 LCL-161; left lower panel) or 4242/MSCV.GFP-Luc, expt #2 (*n*=9 vehicle; *n*=10 LCL-161; right lower panel) were transplanted into syngeneic C57Bl/6 mice (6–12 weeks of age; The Walter and Eliza Hall Institute , Melbourne, VIC, Australia) via tail vein injection. Three days post transplantation, mice were treated with LCL-161 (75 mg/kg) or vehicle control (buffered sodium acetate solution) by oral gavage twice weekly. Mice were killed and autopsied at an end point defined by symptomatic disseminated disease. Kaplan–Meier survival curves are shown. (**b**) Spleen weights at end point for the respective cohorts of mice as described in **a**. (**c**) Percentage of GFP-positive lymphoma cells in peripheral blood of mice receiving either vehicle control or LCL-161 treatment (as per **a**) as assessed by flow cytometry. Peripheral blood was sampled 8 or 11 days post injection with Eμ-*Myc* 107/MSCV.GFP or 4242/MSCV.GFP (expt #2) lymphoma, respectively (as described in **a**). (**d**) Bioluminescence imaging was performed using the Xenogen IVIS platform (Caliper Life Sciences, Waltham, MA, USA) and Living Image software (version 2.5, Xenogen). 100 μl of 1.5 mg/ml D-luciferin substrate (ThermoFisher) was administered by intraperitoneal (i.p.) injection to mice prior to anesthetizing with isoflurane and image acquisition. Representative images of mice treated with vehicle alone or LCL-161 on day 7 post receipt of Eμ-*Myc* 6066/MSCV.GFP-Luc lymphoma cells (as per **a**) are shown. Error bars represent mean±s.e.m. with sample size chosen to ensure adequate power based on previous publications.^13, 14, 15, 16, 17^ *P<0.05; NS, not significant using Student's two-tailed unpaired *t*-test to compare the means of vehicle vs drug-treated groups, and log-rank analysis was used for comparison of survival curves. All mouse experiments were in full compliance with the Peter MacCallum Cancer Centre Animal Ethics Committee.

We therefore assessed the response of the same lymphomas (presented in Figure 1) in an *in vivo* syngeneic transplant setting, utilizing immunocompetent wild-type C57Bl/6 mice. Although we did not necessarily anticipate efficacy (given our *in vitro* findings), unexpectedly worse outcomes were demonstrated for LCL-161-treated mice compared with vehicle controls in the majority of experiments performed (Figure 3a). We observed a reduction in the median survival of mice transplanted with two independently-derived lymphomas (107 and 6066) and treated with LCL-161 (Figure 3a). Either no significant benefit, or a reduction in survival was observed with the third lymphoma (4242) in independent experiments. Premature death was attributable to progressive lymphoma, rather than drug toxicity, as evidenced by equivalent tumor burden (spleen weight) observed in LCL-161-treated mice at end point compared with vehicle-treated mice when they succumbed to lymphoma (Figure 3b). Furthermore, an increased proportion of lymphoma cells in leukemic phase (Figure 3c) and subtly increased lymphoma burden by luciferase reporter imaging was observed in LCL-161-treated mice during treatment (Figure 3d). Thus, LCL-161 treatment can accelerate the progression of Eμ-*Myc* lymphomas *in vivo* resulting in reduced survival in lymphoma-bearing mice.

cIAP1 and 2 have previously been implicated as tumor suppressors for human B-cell malignancies,^18, 19^ and deletion of these genes was shown to promote B-cell survival in the absence of BAFF-R signaling.^18^ Cooperation between MYC and NFκB is also well described in lymphomagenesis^20^ and constitutive NFκB activity is a known driver of mature B-cell malignancies where ‘oncogenic addiction' to NFκB has been reported.^21, 22, 23^ Thus, we posit that LCL-161-mediated degradation of cIAP1 and 2 in the absence of an extrinsic apoptotic response may induce B-cell survival signals including NFκB activation, thus supporting Eμ-*Myc* lymphoma progression *in vivo*, although the suppression of MYC-driven lymphomagenesis by NFκB activation has also been described.^24, 25^

Disease acceleration has been reported with other small molecule therapeutics, most notably BRAF inhibitors causing ‘on-target' acceleration of *RAS-*mutant squamous cell carcinomas^26^ and chronic leukemia.^27^ Although no tumor regressions were documented in the phase 1 LCL-161 study (of 53 patients),^11^ no tumor acceleration was recorded either. As the default interpretation in nonresponders is usually a lack of efficacy, an active detrimental effect (i.e., disease acceleration) may be underappreciated by investigators. Thus, our data provide a cautionary tale to the interpretation of such studies, whereby the investigational agent may promote the progression of certain tumors.

### LCL-161 exacerbates the cytokine storm of LPS challenge

**Figure 4:**
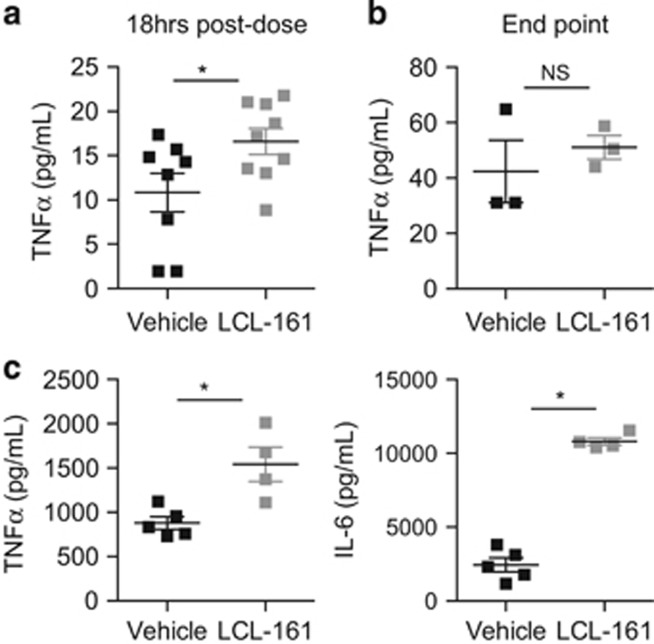
LCL-161 induces cytokine release in lymphoma-bearing mice and exacerbates the response to lipopolysaccharide challenge *in vivo.* (**a**) Mouse serum was interrogated for TNFα using the BD Cytometric Cytokine Bead Array mouse Flex Set and Cell Signaling Master Buffer Set (BD Biosciences, Franklin Lakes, NJ, USA) as per the manufacturer's instructions. C57Bl/6 mice (6–12 weeks of age; The Walter and Eliza Hall Institute of Medical Research) shown were transplanted with Eμ-*Myc* 107/MSCV.GFP lymphoma and treated with two doses of LCL-161 (75 mg/kg; *n*=9) or vehicle (*n*=8 vehicle) in the week following transplantation (described in Figure 3a). Serum was sampled 18 h after the second dose. (**b**) Serum was obtained from mice bearing Eμ-*Myc* 107/MSCV.GFP lymphoma and treated with LCL-161 (*n*=3) or vehicle control (described in Figure 3a; *n*=3) at the time of killing owing to end-stage lymphoma progression and TNFα levels assessed as in **a**. (**c**) Non-tumor-bearing C57Bl/6 mice were pretreated with a single 75 mg/kg dose LCL-161 (*n*=5) or vehicle control (*n*=4) 4 h prior to administration of LPS (E. coli 0127:B8, gamma-irradiated; Sigma-Aldrich) by i.p injection, 4 h prior to collection of serum. TNFα and IL-6 levels in the sampled serum was assessed as per **a**. Error bars represent mean±s.e.m. with sample size chosen to ensure adequate power based on previous publications.^13, 14, 15, 16, 17^ *P<0.05; NS, not significant using a Student's two-tailed unpaired *t*-test to compare the means of vehicle vs drug-treated groups. All mouse experiments were in full compliance with the Peter MacCallum Cancer Centre Animal Ethics Committee.

LCL-161 induces CCL2, IL-10 and TNFα secretion *in vivo.*^11^ Both CCL2 and IL-10 have stimulatory effects on the growth and survival of B-cell lymphomas.^28, 29^ Furthermore, in the recently reported phase I clinical trial of LCL-161 in solid organ malignancies, CRS was described as the main dose-limiting toxicity.^11^ Increases in TNFα and other inflammatory cytokines peaked within 24 h of human LCL-161 administration. Indeed, similar cytokine (TNFα release) was detectable in lymphoma-bearing mice following LCL-161 dosing (Figure 4a), indicating accelerated lymphoma progression occurred in the context of changes to the cytokine milieu. However, the absolute serum TNFα levels were relatively low and not differentially elevated in mice at the time of killing, indicating that premature deaths were unlikely to have resulted from CRS (Figure 4b).

To further evaluate the propensity for IAP antagonists to augment CRS, we next administered LCL-161 to non-tumor-bearing C57Bl/6 mice prior to activation of inflammatory NFκB signaling by injection of a sublethal LPS challenge. The induction of inflammatory cytokines (TNFα and IL-6) in mice challenged with LPS was markedly increased in the presence of LCL-161 (Figure 4c) to the extent that LCL-161 (and not untreated mice) required killing due to endotoxemia 4 h after injection. These data confirm LCL-161 is pharmacologically active in mice at the doses used in lymphoma experiments. Moreover, LCL-161 has the capacity to broadly synergize with inflammatory stimuli and can cooperate in the induction of endotoxic shock *in vivo*.

LCL-161 was recently described as a ‘double-edged sword' in cancer therapy, due to its propensity to induce cytokine release.^30^ We demonstrated similar cytokine release following dosing of lymphoma-bearing mice, although it did not constitute a dose-limiting toxicity. However, LCL-161 augmented a lethal CRS post LPS challenge indicating a further potential clinical risk with SMAC mimetics. Cancer patients are often immunosuppressed due to underlying disease or prior therapies, particularly those with bone marrow dysfunction due to hematological malignancy. Those patients developing bacterial infections while on IAP-inhibitor therapy are likely to be particularly susceptible to septic shock, again calling for clinical vigilance and a low threshold for drug discontinuation during active infection. The notion of this ‘double-edged sword' whereby SMAC mimetics may be detrimental to patients is indeed reinforced by our data, indicating the potential for both disease acceleration and increased susceptibility to endotoxemia. The clinical translation of IAP antagonists requires close monitoring to avoid adverse patient outcomes. In conclusion, we suggest that the use of SMAC mimetics to treat lymphoma should be carefully rationalized according to activity of specific molecular pathways where possible.
